# Infection in asymptomatic carriers of SARS-CoV-2 can interfere with the achievement of robust immunity on a population scale

**DOI:** 10.1099/jgv.0.001684

**Published:** 2021-11-17

**Authors:** Kelvinson Viana, Luis Zarpelon, Andre Leandro, Maria Terencio, Renata Lopes, Caroline Martins, Isaak Silva, Alessandra Sibim, Fábio Marques, Rafael da Silva, Açucena Rivas, Adrieli Souza, Angelo dos-Santos, Sara Torres, Maria Garcia, Rodolfo Giunchetti, Wagner Chiba-de-Castro

**Affiliations:** ^1^​ Universidade Federal da Integração Latino-Americana, Foz do Iguaçu, Brazil; ^2^​ Centro de Controle de Zoonoses de Foz do Iguaçu, Brazil; ^3^​ Fundação Municipal de Saúde de Foz do Iguaçu, Brazil; ^4^​ Universidade Federal de Minas Gerais, Belo Horizonte, Brazil

**Keywords:** SARS-CoV-2, humoral immunity, cell immunity, herd immunity

## Abstract

The severe acute respiratory syndrome coronavirus 2 (SARS-CoV-2) continues to spread worldwide as a severe pandemic, and a significant portion of the infected population may remain asymptomatic. Given this, five surveys were carried out between May and September 2020 with a total of 3585 volunteers in the municipality of Foz do Iguaçu, State of Paraná, a triple border region between Brazil/Argentina/Paraguay. Five months after the first infection, volunteers were re-analysed for the production of IgG anti-Spike and anti-RBD-Spike, in addition to analyses of cellular immunity. Seroconversion rates ranged from 4.4 % to a peak of 37.21 % followed by a reduction in seroconversion to 21.1 % in September, indicating that 25 % of the population lost their circulating anti-SARS-CoV-2 antibodies 3 months after infection. Analyses after 5 months of infection showed that only 17.2 % of people still had anti-RBD-Spike antibodies, however, most volunteers had some degree of cellular immune response. The strategy of letting people become naturally infected with SARS-CoV-2 to achieve herd immunity is flawed, and the first contact with the virus may not generate enough immunogenic stimulus to prevent a possible second infection.

## Introduction

The current pandemic caused by SARS-CoV-2 (Covid-19) is undoubtedly the biggest challenge that the world has been facing in terms of public health, social and economic aspects. In addition to all efforts to combat or minimize its effects, the scientific community has been trying to understand the different clinical forms that the disease presents in people, ranging from asymptomatic conditions to death [1–3].

From the moment that different clinical forms of the disease appeared and their particularities in the manifestations of symptoms and sequelae after recovery, there has been a strong interest in understanding how the adaptive immune system reacts in the control of infection. Some studies have indicated that there are considerable loss of antibodies over time [4, 5]. However, in other studies it is possible to verify that immunity is maintained for at least 6 months, with marked heterogeneity in adaptive immune responses for this new coronavirus [6]. In fact, even totally asymptomatic people can maintain certain levels of anti-SARS-CoV-2 antibodies, but with lower litres when compared to those who had severe cases of the disease [7].

Throughout this pandemic, many epidemiological studies have been carried out, but few with systematic follow-up [8–10]. All of these approaches are extremely relevant in the pandemic context, mainly due to the adoption of proposals based on the term ‘herd immunity’. This strategy was widely commented on, even documented based on data from cities that had a large number of infected people and deaths [11, 12]. Seroprevalence studies show a deeper impact on underserved populations. Recent work done in India showed that the prevalence of anti-SARS-CoV-2 antibodies was higher in slums (54.1%) compared to non-slum areas (16.1%) [13]. Understanding the adaptive responses against SARS-CoV-2 in populations serves as a basis for the design of vaccines, as well as for public policies to combat the virus. Therefore, we conducted serological surveys from May to September, in addition to analysing the cellular and humoral immunity of asymptomatic people who became infected 6 months ago.

## Methods

### Study context

The municipality of Foz do Iguaçu is located in southern Brazil, bordering Paraguay and Argentina. It has 258 823 inhabitants, a demographic density of 414.58 inhabitants per km², an urbanization rate greater than 99 %, and a high human development index (0.751).^4^ The healthcare system consists of 36 service centres, including four hospitals. The total area of the municipality is divided into five health districts. The first confirmed case of Covid-19 in Foz do Iguaçu was classified as imported and occurred on 12 March 2020. The first case of local transmission was reported on 25 March, and community transmission on 7 April. The first death from the disease occurred on 26 April.

School activities were interrupted on 15 March, with a ban on crowded events. As of 17 March, the activities of health clubs, nightclubs, tobacco shops with on-site consumption, and open markets were suspended. Strict health control measures were adopted for shopping malls, supermarkets, bars and restaurants. Public servants were put into home office mode and their working hours were rearranged to avoid travel and crowding. On 19 March, a state of emergency was declared in the municipality for 30 days, and all non-essential activities were shut down on 20 March. On 22 March, the city’s International Bus Terminal and all accommodation facilities are shut down, and on 23 March, Municipal Mass Urban Transport was suspended. These measures lasted until 22 April, when some sectors were allowed to resume their activities, provided that they ensured strict health control measures and used only 30 % of the maximum service capacity. The restricted circulation of older adults and children under 14 years of age was upheld throughout the period.

As of the second week of June, the city experienced an abrupt and sustained growth in the number of cases. On 1 July, a new municipal decree suspended all non-essential economic activities, the operation of parks, squares, sidewalks, weight training equipment and other areas of collective outdoor activities for 14 days.

The calculation of the mean social distancing rate in the municipality used an index created and developed by In Loco (https://inloco.com.br/), a Brazilian technology company that provides intelligence based on location data for cellphones. Anonymous location data is collected from 60 million devices, allowing mobile applications to provide location-aware services while protecting user privacy. The social-distancing index measures the percentage of devices in a given municipality, which remained within a radius of 450 metres from the location identified as home. The index is calculated daily and ranges from zero to one. In the period between the week of confirmation of the first case until 11 July (17 weeks), the mean index for Foz do Iguaçu was 48.63.

### Data collection

We conducted five serological surveys with blood collection from volunteers selected at random to estimate the population that had already come into contact with SARS-CoV-2 in the municipality. The first collection took place 18 days after the first confirmed death by Covid-19 in the municipality, with 924 volunteers. The second collection was carried out 42 days after this death (23 days after the first collection), with 578 volunteers. The third collection was carried out 67 days after the first confirmed death (23 days after the second collection), with 657 volunteers. In the fourth collection, 919 volunteers participated, and it was carried out 20 days after the third collection. In the fifth collection, 507 volunteers participated, and 41 days after the fourth collection. In November, blood was collected from volunteers who presented IgG reagent in June. This specific intervention was not random, as it was of interest to know whether the same people still had detectable antibodies 5 months after the first detection. Only volunteers who agreed to the new blood collection participated in the experiment.

Blocks of the municipality’s urban area were randomly selected for each survey and distributed in the five health districts. We randomly selected a residence in each block and sampled one volunteer in each residence. Blood samples were collected in a vacuum system with a clot activator tube and separation gel, identified and stored in temperature-controlled thermal boxes, remaining at 2–8 °C until being sent to the Vaccine Development Technology Laboratory at the Federal University for Latin American Integration (UNILA). In the laboratory, the samples were centrifuged, and serum stored at a temperature of 20 °C for IgG detection in serum by the ELISA method developed in-house.

### Serological analysis by ELISA

The 96-well microplates (Corning) were sensitized with 1 µg Spike protein or Spike receptor binding domain (RBD)/well and kept at refrigerator temperature for 12 h. After that, the plate was washed three times with buffered saline plus 0.05 % tween 20 (PBS-T). The plates were blocked with 100 µl of blocking solution (Bovine Foetal Serum -SFB+PBS T)/well and kept at room temperature (RT) for 30 min and washed again three times with PBS-T. The serum samples were diluted 1/100 in PBS-T and kept for 30 min at 37 °C, washed three times with PBS-T. Then the conjugated anti-human Ig-G antibodies were added at 1/30 000 dilution (Sigma-Aldrich) and maintained for 30 min at 37 °C, washed three times and added TMB chromogen for 15 min at 37 °C. The reaction was stopped with 35 µl of H_2_SO_4_ at 1M and the readings were carried at wavelength of 450 nm.

Samples were used from patients of different ages and sex RT-PCR positive for SARS-CoV-2, and admitted to the municipal hospital with dates that varied between 10 and 30 days after the onset of symptoms. After standardization, the pool of samples of true positives was established to be used as a positive control in the tests. In addition, different serum were included to analyse the test specificity such as samples from people: vaccinated against H1N1, that acquired Dengue Virus, haemorrhagic dengue, and infected with other coronaviruses (OC43 and 229E variants, which are the most common variants in the region). To establish the cut-off of the test 80 serum samples from real people negative for Covid-19 (samples obtained from a serum bank for different diseases), and from the average of these controls, a correction factor for the test was obtained (factor=2). In this way, the cut-off was established from the average of the negative control (included in each test) plus the correction factor. The tests showed that the in-house ELISA showed a sensitivity of 95 % and a specificity of 99 % for Spike, and a sensitivity of 92 % and specificity of 99 % for RBD. The tests were validated according to RDC 302/2005 of the National Health Surveillance Agency of Brazil (ANVISA).

### Isolation of peripheral blood mononuclear cells (PBMCs)

The cellular immune response of people who had positive IgG for 5 months was accessed by collecting 10 ml of blood samples in heparinized tubes. The PBMCs was obtained as previously described [14]. Briefly, the whole blood volume collected was placed in a mixture of Ficoll-Hypaque (Sigma Chemical; density: 1.077 g ml^−1^) at a 1 : 1 ratio (Ficoll/blood) in sterile polystyrene conical bottom tubes (Falcon, Corning, USA). All samples were centrifuged at 700× for 40 min at 22 °C. The PBMC was collected at the Ficoll-Hypaque interface and transferred to another tube with 40 ml of Falcon sterile 1×PBS containing 10 % FBS. This tube was centrifuged two times at 400× for 10 min at 4 °C. After the supernatant was discarded, the cells were resuspended in 1 ml of cell-culture medium RPMI 1640. Cells were counted in a Neubauer hemocytometer chamber to determine the numbers of monocytes or lymphocytes per millilitre.

### Lymphoproliferation

After counting the cells in the Newbauer chamber, the amount of plated PBMC was 1×10^6^ cells / well in 96-well plates (Corning) in RPMI / SFB 10 % / 37 ° C / 5 % CO_2_. Cultures were maintained for 72 h stimulated with 2 µg of Spike or 2 µg of RBD-S. The lymphoproliferation assay was based on the metabolic reduction of 3-(4,5-dimethylthiazol-2-yl)−2,5-diphenyltetrazolium bromide (MTT) to formazan, and made it possible to evaluate both cell proliferation and viability. Four hours after the end of the incubation period, 20 µl of a MTT solution (2.5 mg ml^−1^) was added to each well (containing 200 µl final volume). After 4 h of incubation and formation of formazan crystals, the supernatant was carefully removed and 200 µl of a 0.04M HCl solution in isopropanol was added to each well. After solubilization of the forming crystals formed by the viable cells’ metabolism of MTT, the plates were read in an ELISA reader at a wavelength of 595 nm.

### Statistical analysis

The statistical analysis was performed with the software GraphPad Prism 5.0 (Prism Software, CA, USA). Data normality was assessed using the Kolmogorov–Smirnov test. The analysis were performed using repeated measures ANOVA. Differences were considered significant at *P* < 0.05.

## Results

### Serological surveys

**Fig. 1.**
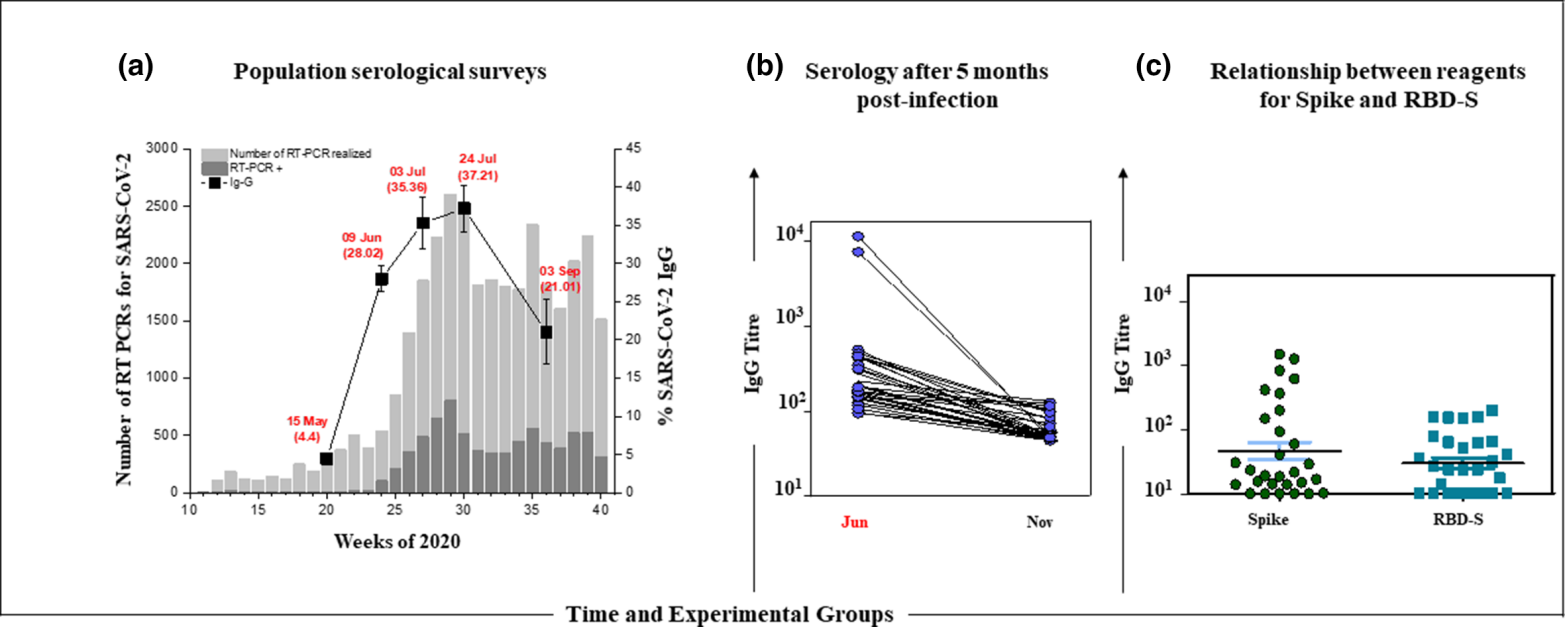
Profile of the anti-SARS-CoV-2 humoral response on a population and individual scale in the city of Foz do Iguaçu, Paraná, Brazil between May and November 2020. (a) Serological surveys carried out between May and September, with 924 volunteers in May, 578 in June, 657 and 919 in July, and 507 in September, totaling 3585 people. Serological data are overlaid with official RT-PCR results for Covid-19 from the municipal health department. (b) Serological analysis of people positive for IgG anti-SARS-CoV-2 in June and retested in the first half of November. (c) Analysis of anti-Spike and anti-RBD-S antibody litres of people positive in June and retested in November 2020.

The total of samples analysed over five interventions was 3581 volunteers; in the first survey, the reagent rate was 4.44 % (CI 95 % 11 472 people), followed by 28.02 % (CI 95 % 72 461 people) in the second, 35.36 % (CI 95 % 91 293 people) in the third, 37.21 (CI 95 % 96211) in the fourth and 21.1 % (CI 95 % 65 427 people) in the fifth survey. These results indicated that after 3 months, on average, on a population scale, there is a 25.01 % reduction in the rate of IgG antibodies in people who came into contact with SARS-CoV-2. In addition, between the third and fourth collection (35.36 and 37.21 %), the indices were within the 5 % error margin, indicating stagnation in seroconversion (Fig. 1a). Throughout the survey period, the social isolation rate was always below what was considered ideal (Fig. S1, available in the online version of this article).

### Humoral analysis after 5 months post-infection

Of the total of 162 reactive people in the month of June, 50 accepted to participate in the new analysis, but 29 attended the blood collections between 9 and 13 November. Of this total, eight (27.5 %) people continued to be positive for Spike and of these, five (17.2 %) were also reactive for the RBD-S (Fig. 1b, c).

### Lymphoproliferative activity after 5 months post-infection

**Fig. 2.**
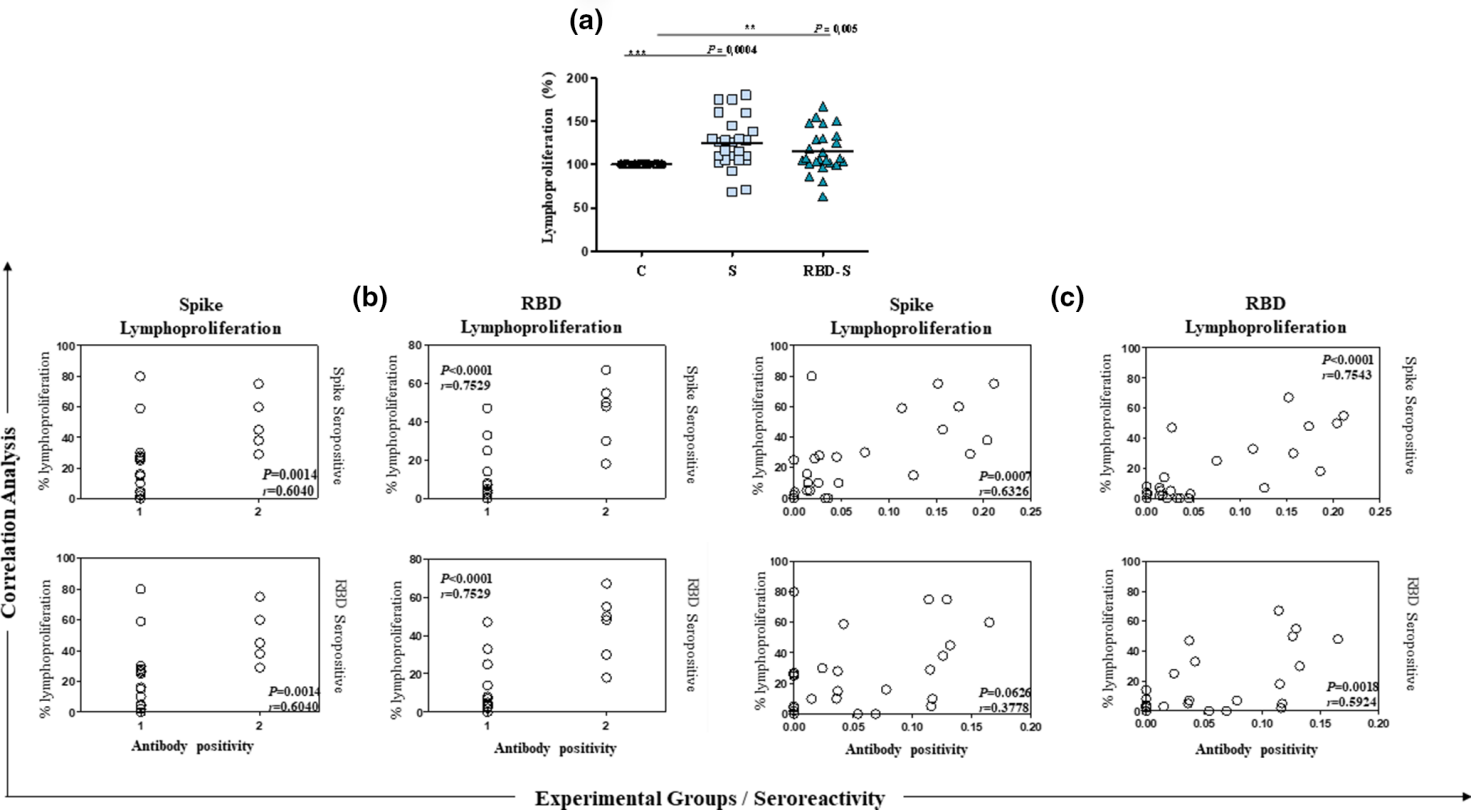
Lymphoproliferative analysis of asymptomatic seropositive carriers for 5 months. (a) Group analysis (Spike and RBD-S) of the total population that accepted to participate in the studies 5 months after soropositive diagnosis for SARS-CoV-2 in June 2020, C: control culture, S: culture stimulated with 2 ug of the Spike protein and RBD-S: culture stimulated with 2 ug of the RBD-S fraction, all maintained for 72 h afterwards MTT assay analyses. The connecting lines between the bars indicate significant differences (*P* < 0.05). (b) Correlation analysis using serological reactivity according to Spike (upper panel) or RBD antigens (bottom panel) and distinct lymphoproliferation stimulus (Spike antigen – left panel or RBD antigen – right panel). The ‘*X*’ axis displayed the serological reactivity as follows: 1=seronegative samples and 2=seropositive samples. (c) Correlation analysis using serological reactivity according to Spike (upper panel) or RBD antigens (bottom panel) and distinct lymphoproliferation stimulus (Spike antigen – left panel or RBD antigen – right panel). The ‘*X*’ axis displayed the optical densities of each analysed sample according to antigenic reactivity by ELISA (Spike or RBD). The correlation indexes are displayed in the graphs showing ‘*P*’ and ‘*r*’ values.

The capacity of the total lymphocytes of people who became infected 5 months ago was analysed by an MTT assay, with their cells stimulated for 72 h with the protein Spike and RBD-S. In this sense, it was found that among the 29 volunteers who agreed to participate in the study again in November, and who had already been reactive in June 2020, the lymphoproliferation rates ranged from zero to 80 %. (Fig. 2a). When analysing the population as a whole, significant differences were found between the two stimuli in relation to the control group. These results may indicate profiles of asymptomatic individuals with different response capacities in the face of a possible second exposure to the virus.

### Correlation analysis

The correlation analysis between serodiagnosis using the spike and RBD-S for lymphoproliferation was *P*=0.8696 and *r*=−0.03461 (Pearson's test). Moreover, the correlation between spike antigen (serological diagnosis) and RBD-S for lymphoproliferation analysis was *P*=0.3218 and *r*=−0.2066 (Pearson's test) (Fig. 2b). The serological reactivity using the Spike at June (original antibody level) and the same antigen analysed in November (*P*=0.5960 and *r*=0.1606) or the RBD-S (*P*=0.6662 and *r*=0.09074) resulted in the absence of correlation in the Pearson correlation test (Fig. 2c).

## Discussion

In the present study, we sought to analyse the dynamics of anti-SARS-CoV-2 seroconversion, through five serological surveys in the municipality of Foz do Iguaçu, Paraná, Brazil, between the months of May to September 2020. In addition, to re-analyse the positives in the month of June in order to verify if the humoral and cellular immunity was maintained. Our results indicated that surveys carried out systematically help a lot in understanding the maintenance or not of the levels of antibodies against the new coronavirus.

Contrary to some ideas launched at the beginning of this pandemic, including adopted as a public policy to combat SARS-CoV-2 by some governments, herd immunity from natural infection is fanciful, as the population is unable to achieve robust levels of immunity above 70 % and keep them for long periods. And in this context, we are witnessing a new wave of cases in different countries such as Belgium, Germany, France, Spain, Ireland, Czech Republic [11, 12, 15] as well as in Brazilian cities that suffered a lot with several deaths and now the moving average of active cases has increased again. As mentioned in another study [5], IgG are lost on average 3 months after infection, and symptomatic groups are able to maintain better levels compared to asymptomatic ones. Our data showed that the seroconversion rate stagnated between the third (35.36 %) and fourth survey (37.21 %), with a reduction to 21.1 % in September. However, the reduction in the population-scale IgG rate was 25 % in 3 months (Fig. 1a). On the other hand, people with mild symptoms lost only 16 % after 4 months of infection [16]. This information directly complicates decision-making when facing the pandemic, in addition to serving as an alert for mass immunization strategies, as well as the time for vaccine boosters. More recently, a study released by the Butantan Institute of Brazil, but not yet published, confirmed that herd immunity can be acquired by mass immunization. The study showed that the pandemic virus can be controlled with vaccination. Symptomatic cases of Covid-19 have dropped by 80 % since the start of the mass vaccination, related hospitalizations fell 86 %, and deaths plummeted 95 % [17].

An interesting piece of data in this study shows that the curve of positives by serology rises before the number of people positive by RT-PCR. Indicates that the number of people infected was greater than the official results. This was because the molecular testing methodology was only for those with Covid-19 symptoms. This strategy was adopted because there were no resources to test asymptomatic individuals, and it was the method widely adopted in different countries. However, studies that performed larger tests indicated that up to 70 % of positive individuals had mild symptoms or no symptoms [18–20], and in the United States the rate of infected could be up to 20 times higher than the number of cases confirmed [21].

From the blood tests of those who became infected 5 months ago, we found that the reduction in seroconversion rates for the Spike protein continues to be marked (eight reagents out of 29). When we verified the presence of Ig-G for RBD-S, which is more interesting to block viral anchorage at the ACE2 receptor, only 17.2 % of people maintained the immunoglobulin. In fact, immunity to SARS-CoV-2 is not limited to antibodies, although they are fundamental in controlling infection. It is known that symptomatic patients followed for 100 days after the onset of infection have different dynamics in seroconversion. Virus-specific IgG decayed substantially in most individuals, whereas a distinct subset had stable or increasing antibody levels in the same time frame despite similar initial antibody magnitudes [22]. On the other hand, antibodies against SARS-CoV-2 spike and RBD declined moderately over 8 months, in a report that mostly involved people with mild disease [23]. In another study, it was found that reinfections by natural infection occur for all four seasonal coronaviruses, suggesting that it is a common feature for all human coronaviruses, including SARS-CoV-2 [24]. This is in agreement with our results, as between June and September there was a loss of antibodies in around 25 % of the population, in addition to different forms of lymphocyte reactivity to viral antigens.

The RBD-S fragment is a domain that mediates the binding of the virus to ACE2 and has much more affinity for this receptor compared to SARS-CoV-1 [25]. Thus, the presence of this class of antibodies in populations affected by the virus would be much more interesting in the epidemiological context, due to the capacity for viral neutralization. Our results showed that these antibodies are present at the lowest rate in people who had antibodies 5 months after infection. On the other hand, in a study in the United Kingdom, it was found that the presence of anti-Spike antibodies provided protection against reinfections for most people in the 6 months following infection [26]. However, in another analysis of the dynamics of neutralizing antibody litres in eight convalescent patients with Covid-19, four patients showed decreased neutralizing antibodies approximately 6–7 weeks after illness onset [27]. Furthermore, in cases of convalescent patients, it has been suggested that plasma from these patients (for therapy) should be recovered quickly after the resolution of symptoms, because there would be a reduction in IgG and IgA levels between 6 and 10 weeks after the onset of symptoms [28]. However, even after months of infection, although there is a general decline in antibodies, IgG +B cells remain stable [29, 30]. In addition, broad and strong memory CD4 +and CD8+T cells can be found in >90 % of convalescent Covid-19 patients [31, 32]. Comforting data indicate that the humoral response in immunized with mRNA vaccines (mRNA-1273 or BNT162b2) and individuals recovered from natural infection show similarities between antibody litres and neutralizing capacity [33].

Cellular immunity is as important as humoral immunity in controlling coronavirus infection, both complement each other [34, 35]. In different studies trying to elucidate these issues, it is verified that, although most people (symptomatic or asymptomatic) lose some amounts of antibodies, we do not have large numbers of cases of reinfections [[23, 36, 37]]. However, there are currently hundreds of cases under investigation, and reinfections are certainly not the norm, but it is increasingly common to report people who have been positive on RT-PCR twice since the beginning of the pandemic. It is understandable that even with low antibody litres, cellular memory immunity is containing reinfections with more severe conditions [6, 38, 39]. Five months after infection, we found that the total lymphocytes of most asymptomatic people were able to proliferate after stimulation with at least one of the antigens (Spike or RBD-S) (Fig. 2a). However, we did not observe a correlation between the antibody level in June and lymphoproliferation at 5 months, nor a correlation between the original antibody level and the persistence of the antibody level after 5 months (Fig. 2b, c). In part, this may help explain few cases of reinfection within 5 or 6 months post-infection. In addition, asymptomatic people tend to have a less robust immune response compared to symptomatic ones, and this could be a concern in an epidemiological context. In this context, a possible susceptibility to new reinfections needs to be analysed in the long term, and whether these reinfections will be characterized by asymptomatic or symptomatic clinical conditions, with analysis of viral load. This last point deserves greater attention and surveillance, especially due to the emergence of new variants with greater infective capacity [40–42].

In general, our study indicates that on a population scale, the anti-SARS-CoV-2 seroconversion rate is reduced by around 25 % in people who had asymptomatic infection within 3 months of infection, and that this is a risk factor in public policies based on the idea of herd immunity. Furthermore, 5 months post-infection, only a small number of people are able to maintain potentially neutralizing antibodies, and the cellular immunity of these asymptomatic people can range from zero to robust. Everything indicates that it is this cellular response, in different degrees of reactivity, that manages to prevent a variety of reinfections. However, it is necessary to analyse this pattern of immunity in the long term.

## Supplementary Data

Supplementary material 1
