# Supplementary material for: Infection in asymptomatic carriers of SARS-CoV-2 can interfere with the achievement of robust immunity on a population scale
Source: J Gen Virol. 2021 Nov 17;102(11):001684. doi: 10.1099/jgv.0.001684 (PMC8742991; doi:10.1099/jgv.0.001684)
Supplement: Supplementary material 1 [file jgv-102-1684-s001.pdf]

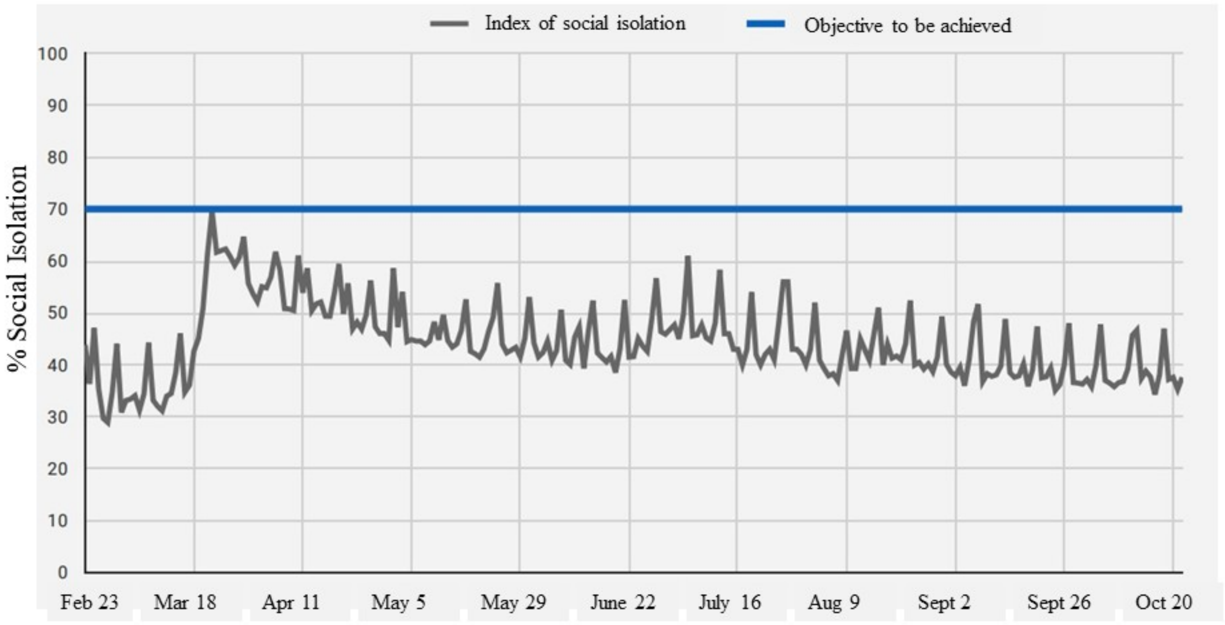

Supplementary Figure 1(S1). **Social distancing rates in the Municipality of Foz do Iguaçu, Paraná, Brazil between February and October 2020**. Data were adapted from the official website https://inloco.com.br/. The blue line indicates the objective that would be considered ideal to reduce viral spread, and the gray line indicates the variations in social isolation achieved in the period.
